# Kind Words and Suggestions

**Published:** 1873-04

**Authors:** J. A. Hanks


*Kind Words and Suggestions.*

North Carolina.—"I am much interested in the permanent success of the Record. I was a subscriber when it had the name of the *Georgia Medical Companion*, and, although the name is changed, my feelings and hopes for its success are not in the least abated. It is a Southern medical journal with it pages open to the worthy of every section. I love it, and have done, and will continue to do, all in my power to advance its interest. I think you will see that I have sent you the names of six new cash subscribers, and this with no great trouble. I feel that every subscriber can (with as little trouble) do as much, and believing all must feel as I do in regard to the merits of the Record, and the necessity of sustaining just such a journal, I hope you will allow me, as one who has for more than thirty years felt its need, to call upon every subscriber to try and see what they can do. Let every one select five or more friends, and request them, verbally or by letter, to take the RECORD at least one year on trial. I have secured six new subscribers, and pledge myself for at least two more. Come up, gentlemen, and work for the journal of the profession. Let each present subscriber send in from two to five new cash subscribers, and I am satisfied that the RECORD will soon be the best, most useful, and the cheapest medical journal ever published in this country. J. A. HANKS, M.D."

We are not personally acquainted with the writer of the above, and for that reason we thank him the more for the kind interest he has manifested in extending the circulation of the RECORD. We publish the letter to show our readers what is being done for the RECORD by some of its friends, and the high appreciation our efforts to serve the profession are meeting from many quarters. We trust all our subscribers will go to work on the line indicated in the letter above. If they will, unitedly, put their energies in the work of extending the circulation of the RECORD, we will promise to enlarge it at the earliest practicable day.

